# Case report: Ectopic pregnancy in the interstitial part of the fallopian tube

**DOI:** 10.3389/fsurg.2023.1197036

**Published:** 2023-07-03

**Authors:** Diana Bužinskienė, Monika Mačionytė, Darius Dasevičius, Mindaugas Šilkūnas

**Affiliations:** ^1^ Faculty of Medicine, Vilnius University, Vilnius, Lithuania; ^2^ Center of Obstetrics and Gynecology, Institute of Clinical Medicine, Faculty of Medicine Vilnius University, Vilnius, Lithuania; ^3^ National Center of Pathology, Affiliate of Vilnius University Hospital Santaros Klinikos, Vilnius, Lithuania

**Keywords:** interstitial pregnancy, salpingectomy, laparoscopy, laparotomy, methotrexate

## Abstract

Ectopic pregnancy remains one of the most common causes of pregnancy-related death in the first trimester. 2.4% of ectopic pregnancies occur in the interstitial part of the fallopian tube. As the symptoms of this condition are non-specific and the localization is associated with a higher risk of bleeding, early diagnosis of interstitial pregnancies is important, based not only on clinical symptoms, but also on additional diagnostic methods. Early diagnosis leads to better treatment-related outcomes. We report a 32-year-old female patient who came to the emergency department because of pain in the lower abdomen and right iliac region and bloody vaginal discharge. During palpation of the abdomen, the pain was localized in the lower part of the abdomen. Human chorionic gonadotropin (hCG) was significantly increased in biochemical tests. Transvaginal ultrasound examination of internal genital organs, abdominal and pelvic computer tomography (CT) were per-formed. An ectopic pregnancy was suspected. Thus, the patient was hospitalized in the gynecology department for surgical treatment. A laparoscopy was performed and an ectopic pregnancy was diagnosed in the interstitial part of the right fallopian tube and in the right uterine corner, which led to right salpingectomy and right uterine angle resection. Thus, interstitial pregnancy is a rare and life-threatening gynecological condition due to the higher risk of bleeding compared to other ectopic pregnancies. However, appropriate diagnosis based on clinical signs, transvaginal ultrasound findings and hCG levels in the blood ensures early diagnosis of interstitial pregnancy, which leads to the choice of medical treatment with methotrexate or minimally invasive surgical techniques.

## 1. Introduction

An ectopic pregnancy (EP) is a condition in which a fertilized egg is implanted outside the uterine cavity. The prevalence of EPs has increased from 0.5% to 2% since 1970 and remains the most common cause of pregnancy-related death in the first trimester (1, 2). Implantation usually takes place in the ampullary part of the fallopian tube, and less often intra-abdominally or in the cervix. In 2.4% of cases, the interstitial part of the fallopian tube is implanted (3). In EPs, clinical signs such as lower abdominal pain, abnormal uterine bleeding and amenorrhea are not specific and may signal other pregnancy-related conditions such as miscarriage (4). The incidence of EP among women who present with genital bleeding in the first trimester, or lower abdominal pain, or both, is between 6% and 16% (1). Symptoms of interstitial pregnancies occur later compared to other EPs in the fallopian tube. For these reasons, diagnosis is often delayed, leading to an increased risk of bleeding and mortality (5). However, based on clinical signs, transvaginal ultrasound (TVUS) examination results, serum human chorionic gonadotropin (hCG) and progesterone measurement, the diagnosis is more accurate and can be made earlier, leaving an option for systemic treatment with methotrexate or laparoscopic uterine angle resection and salpingectomy. In case of ectopic mass rupture and hemodynamic instability, laparotomy is performed (6, 7). In this article, we review the literature on the etiology, risk factors, diagnosis and treatment of interstitial pregnancy and present a clinical case.

## 2. Case presentation

**Figure 1:**
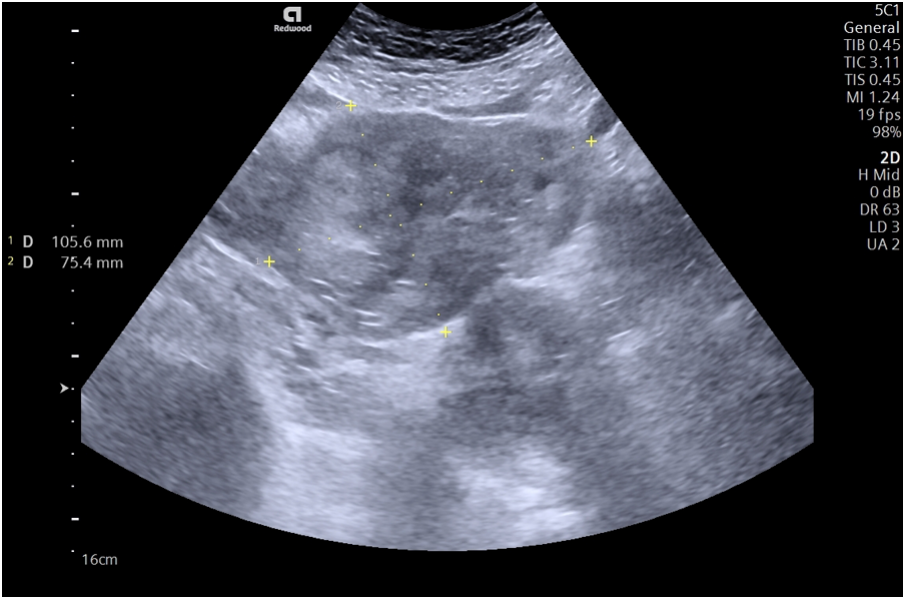
A transvaginal ultrasound showing a formation of heterogeneous mass in the region of the right ovary and fallopian tube.

A 32-year-old caucasian female patient presented to the emergency department of the hospital with pain in the lower abdomen and right iliac region and bloody vaginal discharge. Last menstrual period started one week earlier than usual. Based on her medical history, the patient had one caesarean section (CS), no gynecological or other chronic diseases. She was hemodynamically stable, arterial blood pressure (BP)—175/96 mmHg, heart rate (HR) 82—bpm, rhythmic heart tones. The patient was conscious, Glasgow Coma Scale (GCS) was 15. Body temperature was 36.9°C. There were no rashes on the skin, no enlargement of peripheral lymph nodes, no oedema. Urination and defecation were normal. During abdominal palpation, the pain was localized in the lower abdominal part without muscle tension. The patient rated her abdominal pain as 6 out of 10 on a visual analogue scale (VAS). Laboratory tests showed normal number of leucocytes (WBC)—5.59*10*9/L (normal range, 4.0–9.8), normal level of hemoglobin (Hb)—120 g/L (normal range, 117–145), normal hematocrit (Hct) count—36.8% (normal range, 36–42), slightly elevated CRP—12.4 mg/L (normal range, ≤5) and elevated hCG—998 U/L. On vaginal examination, there was an abnormal uterine bleeding, the cervix was epithelialized normally, the uterus was normal in size, sensitive to palpation, the right ovary and fallopian tube was painful. Transvaginal ultra-sound (TVUS) was performed—the uterus and left ovary were intact, no pregnancy was observed in the uterine cavity, and a heterogeneous mass of 105 × 75 mm was observed in the area of the right ovary and fallopian tube (Figure 1).

**Figure 2:**
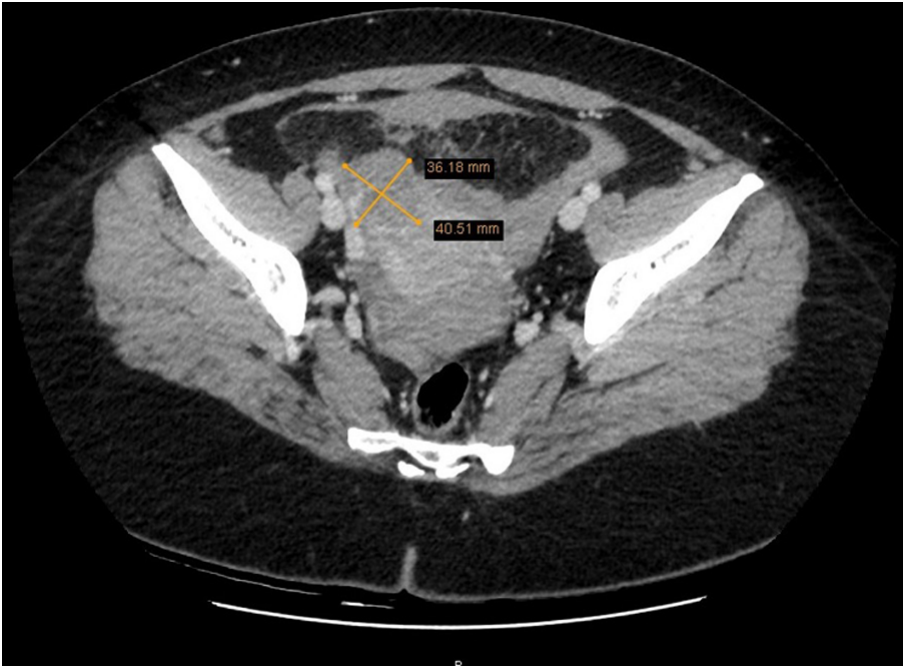
Abdominal and pelvic computed tomography showing a 35 mm heterogeneous vascularized formation in the projection of the right ovary.

The patient was consulted by an abdominal surgeon, no acute abdominal surgical pathology was found. Due to unclear diagnosis, abdominal and pelvic computed tomography (CT) was performed. CT scan during native and post I/v contrast in the arterial and port venous phases showed no pathological changes of the uterus, a 35 mm heterogeneous vascularized mass was visualized in the right ovarian projection, with a fragment of the artery widened to 4.6 mm (Figure 2).

**Figure 3:**
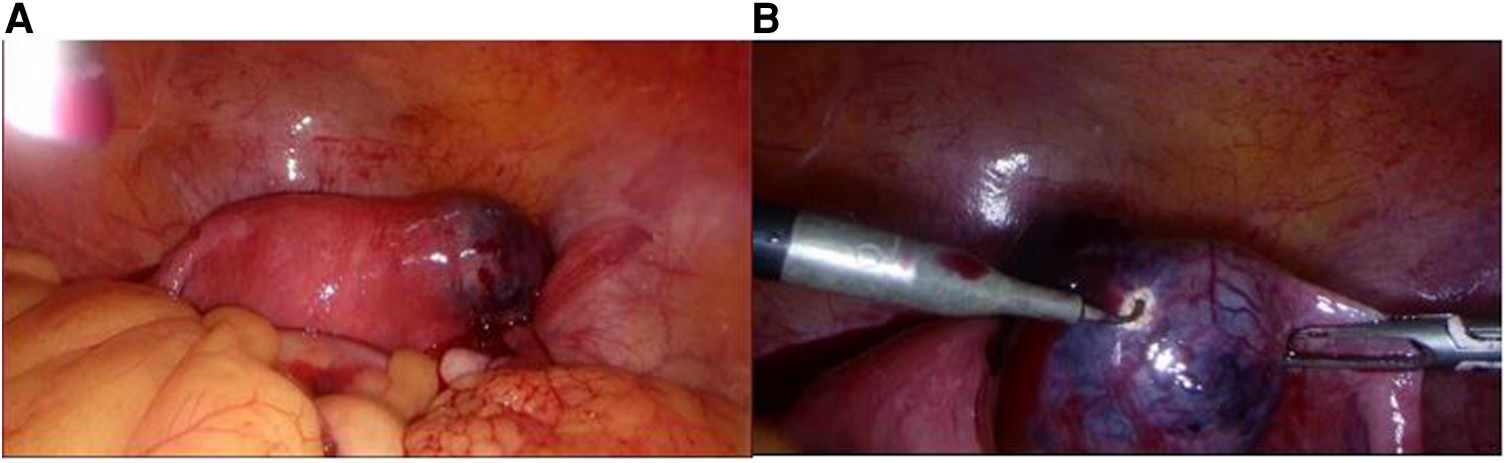
(**A**) Intraoperative view of a cyanotic mass in the right corner of the uterus. (**B**) A closer intraoperative view of the cyanotic mass in the right corner of the uterus.

No active bleeding was observed, in the rectouterine pouch of Douglas native hyperdense (about 65 HV) masses—clots up to 56 × 26 mm in size—were visible in contact with the uterine appendages of both sides. In the abdominal and pelvic cavities, a small amount of free hemorrhagic fluid (about 50–60 HV) was visible. EP was suspected. She was admitted to the gynecology department for surgical treatment of a suspected EP. Preoperative antibiotic prophylaxis with cefazolin 1 g intravenously was administered. During the laparoscopy, a cyanotic mass of about 3–4 cm in size was observed in the right corner of the uterus, including the interstitial part of the right fallopian tube (EP), the mass was not ruptured, without bleeding (Figure 3).

The right fallopian tube was anatomically and functionally altered, both ovaries and the left fallopian tube were without visible changes. A small amount of blood (about 50 ml) was observed in the pouch of Douglas. A right tubectomy with excision of the right uterine angle was performed. The uterine angle was sutured with a few polyglactin sutures.

**Figure 4:**
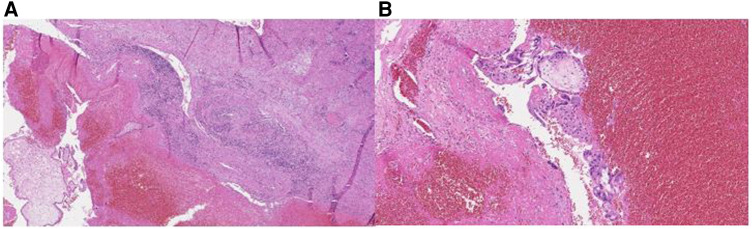
Figure 4 (**A**) Immature slightly edematous chorionic villi (on the left), surrounded by erythrocytes and fibrine (in the center), with muscular wall of the isthmic part of the fallopian tube (on the right). H&E, 4x. (**B**) Closer view at the chorionic villi (composed of cytotrophoblast, sintytiotrophoblast), floating in blood clots background. H&E, 10x.

The fallopian tube (6 cm in length), with angular part of the uterus (3 × 2 × 1 cm of dimentions) was sent to pathology department. Immature slightly edematous chorionic villi (composed of cytotrophoblast, sintytiotrophoblast) and blood clots in the lumen of the interstitial part of fallopian tube were identified by the pathologist of the sent specimen. Ampullar, isthmic part and fimbriae were intact (Figure 4).

Post-operatively, she was given analgesics, intravenous fluid infusions, and thrombo-embolism prophylaxis with Bemiparin sodium 2,500 IU subcutaneously for 7 days. The patient felt well after surgery. The next day, in good overall condition, she was discharged home for further outpatient treatment.

## 3. Discussion

EP is a common cause of morbidity and mortality in women, with an incidence of 2%–3% of all pregnancies and the mortality rate between 2% and 2.5% (8). Between 1970 and 1990, countries such as the United States of America, Norway, Sweden and the United Kingdom experienced a marked increase in the prevalence of EPs, and since 1990 the incidence has started to decline. Worldwide, the incidence of EP is 10.2% lower be-tween 1990 and 2019. Such epidemiological changes in dynamics can be attributed to the spread of chlamydia infection and, subsequently, to effective infection prevention (9).

Ectopic implantation in the fallopian tube is the most common and accounts for 90% of all EP. Implantation usually occurs in the ampulla of the fallopian tube, less frequently in the isthmic part or in the fimbriae. Other localizations of EP are rare or very rare: in the interstitial part of the fallopian tube, ovary, intra-abdominal cavity, cervical canal or in the site of the cesarean scar (3, 10, 11).

The risk factors for EP are categorized as high, medium and low risk. High risk factors include previous EP, previous tubal surgery and/or pathology, tubal ligation, exposure to diethylstilbestrol, use of intrauterine device. Intermediate risks include in-fertility, fertility treatment, pelvic inflammatory disease, previous sexually transmitted infections (STIs) due to chlamydia or gonorrhea, and tobacco smoking, while low risks include previous pelvic surgery and vaginal washing. Despite a wide range of risk fac-tors, 35%–50% of women diagnosed with EP have no risk factors associated with EP (11).

EP in the fallopian tube is caused by impaired migration of the fertilized egg in the fallopian tube. If the movement of the fertilized egg in the fallopian tube is slowed down, the embryo may implant in the fallopian tube before it reaches the endometrial cavity (12). In interstitial pregnancies, the implantation of the gestational sac takes place in the uterine cornea or in the proximal part of the fallopian tube. This is a relatively thick area, on average 0.7 mm in diameter and 1–2 cm in length. Compared to the distal part of the fallopian tube, implantation in this area tends to be more prone to rupture. For these reasons, an interstitial pregnancy may be asymptomatic until the 7th to 16th week of gestation, at which time rupture of the fallopian tube can lead to life-threatening hemorrhage due to the abundant blood supply from the uterine and ovarian vessels at this site (13).

Diagnosis of an interstitial pregnancy is based on clinical history, serum hCG concentration, TVUS and sometimes serum progesterone measurement (14). The most common symptoms of interstitial EP are abdominal pain and light abnormal uterine bleeding in the first trimester of pregnancy and are not specific to this condition (6). Women who experience these symptoms, i.e., pain in the lower abdomen, with or without genital bleeding, should have a quantitative hCG blood test to detect or rule out pregnancy. Despite the fact that women with EP usually have lower hCG concentrations than women with intrauterine pregnancies, a single method of serum hCG measurement cannot determine the location of the pregnancy. Moreover, EP can present with increasing, decreasing or flat curve of hCG concentrations, so serial measurement of hCG is the most useful for confirming fetal viability, but not for diagnostics of EP and should be considered in conjunction with the results of serum progesterone levels. Measuring serum progesterone levels is a potentially useful addition to serum hCG, as it is stable in the first trimester and independent of gestational age (4). To identify the location of the pregnancy, it is important to perform radiologic imaging tests. TVUS is the main radiology imaging method in the diagnosis of EP and can aid in the early detection (14). A research study by Tulandi and Al-Jaroudi presented diagnostic and management of 32 cases of interstitial pregnancy. The study found that a gestational sac was identified in 40.6% of patients and a hyperechoic mass in the cornual region in 25% of patients. Out of the 32 patients, 71.4% were diagnosed with interstitial pregnancy. The sensitivity and specificity of the ultrasound were reported as 80% and 99%, respectively. Additionally, laparoscopy has been suggested as another diagnostic tool due to its ability to aid in both diagnosis and treatment (5). The presence of a mass in the region of the uterine appendages which is seen to move in-dependently of the ovary, with a yolk sac or an embryo, or both, is one of the criteria for the diagnosis of an EP. Other criteria suggestive of EP include the presence of an inhomogeneous mass in the region of the uterine appendages, moving independently of the ovary, or an extrauterine empty gestational sac (15). The latter has a positive predictive value of only 80%, as it requires differentiation from other pelvic structures (paratubal cyst, corpus luteum, hydrosalpinx, endometrioma, bowel). Diagnosis of EP is unlikely after visualization of pregnancy in the uterine cavity, although the possibility of heterotopic pregnancy also remains (16). Accurate diagnosis of EP is essential, as a misdiagnosis of an EP can lead to the termination of a life-compatible pregnancy in the uterus. Conversely, failure to diagnose an EP increases the risk of complications such as life-threatening intra-abdominal bleeding (17).

With the rapid development of TVUS testing and the increasing availability of sensitive hCG testing, most interstitial pregnancies are diagnosed before rupture occurs. For these reasons, medical or surgical treatment is possible (7). Local and systemic methotrexate is the most widely studied and used drug for the treatment of interstitial pregnancies in young, hemodynamically stable women in the absence of ectopic mass rupture. In addition, some authors recommend local injection of methotrexate or potassium chloride into the gestational sac. However, this method of treatment re-quires special equipment and trained personnel, and is less available and more expensive than systemic methotrexate injection (18). The optimal surgical approach for interstitial ectopic pregnancy remains uncertain and lacks a consensus. Laparoscopic surgery is the standard surgical treatment for EP and interstitial pregnancies in hemodynamically stable patients, as it is associated with lower intraoperative blood loss, shorter operative time, less postoperative pain, faster postoperative recovery, and a shorter hospital stay (19, 20). Laparotomy for uterine angle resection to preserve fertility or rarely hysterectomy performed in the case of late diagnosis of interstitial pregnancy and to ensure hemostasis in the case of massive intraabdominal hemorrhage (21). Laparoscopic surgery includes uterine angle resection and salpingectomy (20). However, conservative procedures such as cornuostomy instead of cornual resection is an increasingly common approach (22). Various endoscopic techniques such as electrocauterization, the Endoloop application, or the encircling prior to conceptus evacuation are discussed to reduce the risk of bleeding (23). Moon et al. detailed their use of either Endoloop in 15 patients or an encircling technique in 3 patients before the cornuostomy incision to extract the products of conception. They concluded that these methods were both simple and safe, effectively preventing major hemorrhage (24).

The clinical case presented in this article was characterized by diagnostic difficulties. The patient’s hCG did not correspond to the clinical picture, and radiologic imaging results were not specific for this threatening pathology. Furthermore, the rarity of the condition has led to little clinical experience. Owing the rarity of this clinical case, we wish to highlight the issues in the diagnosis of this insidious condition and to present one of the management methods for treatment of interstitial pregnancy.

## 4. Conclusions

Interstitial pregnancy is a rare but life-threatening condition due to the risk of bleeding after rupture of an ectopic mass. Although high, medium and low risk factors have been identified, up to half of interstitial pregnancies may not have any risk fac-tors. Symptoms of this condition, such as lower abdominal pain and genital bleeding, are not specific and, due to the implantation site of interstitial pregnancies, occur later than in other EP. Thus, the diagnosis of interstitial pregnancy is based on clinical symptoms and serum hCG changes together with TVUS results. By combining different diagnostic approaches, the diagnosis of interstitial pregnancy is made earlier, allowing medical treatment with methotrexate or minimally invasive surgical approaches such as laparoscopic removal of the uterine angle and salpingectomy. In the case of late diagnosis, less conservative surgery, such as laparotomy resection of the uterine angle or removal of the uterus due to heavy bleeding, is used because of increased risk of com-plications.

## Data Availability

The original contributions presented in the study are included in the article, further inquiries can be directed to the corresponding author.
